# Determinants of HIV-1 virologic suppression in HIV-associated tuberculosis in Brazil

**DOI:** 10.1590/0074-02760250090

**Published:** 2026-07-10

**Authors:** Felipe Ridolfi, Gustavo Amorim, David W Haas, Maria Arriaga, Cody Staats, Marcelo Cordeiro-Santos, Afrânio L Kritski, Marina C Figueiredo, Bruno B Andrade, Timothy R Sterling, Valeria C Rolla, the Regional Prospective Observational Research in Tuberculosis (RePORT)-Brazil Consortium, Aline Benjamin, Flavia M Sant'Anna, Jamile Garcia de Oliveira, João Marin, Adriana Rezende, Anna Cristina Carvalho, Michael Rocha, Betânia Nogueira, Alexandra Brito, Renata Spener, Megan Turner

**Affiliations:** 1 Vanderbilt University Medical Center, Department of Medicine, Division of Infectious Diseases, Nashville, TN, USA; 2 Fundação Oswaldo Cruz-Fiocruz, Instituto Nacional de Infectologia Evandro Chagas, Rio de Janeiro, RJ, Brasil; 3 Vanderbilt University Medical Center, Department of Biostatistics, Nashville, TN, USA; 4 Fundação Medicina Tropical Dr Heitor Vieira Dourado, Manaus, AM, Brasil; 5 Universidade do Estado do Amazonas, Manaus, AM, Brasil; 6 Universidade Federal do Rio de Janeiro, Faculdade de Medicina, Rio de Janeiro, RJ, Brasil; 7 Multinational Organization Network Sponsoring Translational and Epidemiological Research Initiative, Salvador, BA, Brasil; 8 Faculdade de Tecnologia e Ciências, Salvador, BA, Brasil; 9 Fundação Oswaldo Cruz-Fiocruz, Instituto Gonçalo Moniz, Laboratório de Pesquisa Clínica e Translacional, Salvador, BA, Brasil; 10 Universidade Salvador, Salvador, BA, Brasil; 11 Escola Bahiana de Medicina e Saúde Pública, Salvador, BA, Brasil; 12 Clínica de Saúde Rinaldo Delamare, Rio de Janeiro, RJ, Brasil; 13 Secretaria de Saúde de Duque de Caxias, Rio de Janeiro, RJ, Brasil; 14 Instituto Brasileiro para Investigação da Tuberculose, Fundação José Silveira, Salvador, BA, Brasil

**Keywords:** TB/HIV, genetic polymorphisms, HIV-1 treatment

## Abstract

**BACKGROUND:**

Tuberculosis/human immunodeficiency virus (TB/HIV) coinfection is associated with advanced HIV disease and variable responses to antiretroviral therapy (ART).

**OBJECTIVES:**

We examined whether baseline HIV severity markers, ART regimes, or human genetic variants influenced HIV-1 virologic suppression in HIV-associated TB.

**METHODS:**

We included TB/HIV participants from Regional Prospective Observational Research in Tuberculosis (RePORT)-Brazil study, who received standard TB therapy and antiretroviral treatment. The primary endpoint was HIV-1 virologic suppression (≤ 1,000 copies/mL); Baseline characteristics, viral load (VL), CD4 cell count, timing of ART initiation, and ART regimens were included. We genotyped *UGT1A1* (rs887829; integrase strand transfer inhibitor-related) and *CYP2B6* [rs3745274, rs28399499, rs4803419; efavirenz (EFZ)-related]; all have defined normal, intermediate, and slow genotypes. Genotyping was performed by MassARRAY iPLEX Gold; Kaplan-Meier curves compared time-to-suppression with log-rank tests; Cox proportional hazards models estimated hazard ratios.

**FINDINGS:**

Among 194 participants, 68% (n = 132) achieved virologic suppression (≤ 1,000 copies/mL). Median time-to-suppression: 84 days [95% confidence interval (CI): 42-125]. Participants with higher baseline viral load (BVL) (≥ 5 log_10_ copies/mL) had delayed suppression compared with those with lower VL (< 5 log_10_; log-rank χ² = 75.9; p < 0.001). Individuals with CD4 ≤ 200 cells/µL suppressed more slowly than those with CD4 > 200 cells/µL (log-rank χ² = 29.6; p < 0.001). Participants starting ART before TB treatment achieved suppression faster than ART-naïve individuals (32 vs. 147 days; log-rank χ² = 48.5; p < 0.001). Higher BVL was associated with reduced hazard of suppression [adjusted hazard ratio (aHR) = 0.67; 95% CI: 0.61-0.75], while higher baseline CD4 count increased the hazard of suppression (per 100 cells/µL: aHR = 1.11; 95% CI: 1.01-1.21). ART-naïve status was associated with lower hazard of suppression in univariate analysis (Hazard Ratio = 0.51; 95% CI: 0.36-0.72) but not after adjustment. ART regimen class and pharmacogenetic metabolizer profiles were not significantly associated with virologic suppression.

**MAIN CONCLUSIONS:**

BVL and CD4 count were the strongest determinants of virologic suppression in TB/HIV patients. Suppression rates were low, and neither ART regimen nor pharmacogenetic profiles significantly influenced the likelihood of suppression.

Human immunodeficiency virus (HIV) infection is a risk factor for the development of tuberculosis (TB)^1^ ^,2^ ^,3^ and treatment of both disease is of high priority for TB/HIV co-infection management.^4^ ^,5^ However, TB and HIV regimens have drug-drug interactions and are also associated with toxicity,^6^ which can impact the outcome of TB/HIV treatment in two ways: subtherapeutic concentrations can result in treatment failure and drug resistance, and supratherapeutic concentrations may be associated with treatment toxicity.^7^ ^,8^ ^,9^ Moreover, the serum levels of some TB and HIV drugs can be influenced by single nucleotide polymorphisms (SNPs) of genes involved in the metabolism of these drugs.^10^ ^,11^ Of the 25 antiretroviral therapy (ART) drugs approved by the Food and Drug Administration (FDA), nine (36%) are known to have SNPs associated with plasma exposure and/or side effects.^12^,^13^,^14^,^15^,^16^,^17^,^18^,^19^,^20^ The proposed mechanisms of TB and HIV drug interactions are mainly related to substrate activity, particularly inhibition or induction of the hepatic system of cytochrome P450. Considering non-nucleoside reverse transcriptase inhibitors and integrase strand transfer inhibitors, the inducers of the enzymatic system (*e.g.*, normal metabolizers) decrease serum drug concentrations, while inhibitors (*e.g.*, slow metabolizers) increase the concentration.^7^

Rifampicin, the backbone of first-line TB regimens, is a potent inducer of hepatic enzymes and drug transporters, leading to reduced plasma concentrations of several antiretrovirals and raising the risk of impaired virological suppression.^21^ ^,22^ Despite these concerns, a systematic review and meta-analysis found that HIV-infected patients initiating ART while on TB treatment achieved virological suppression rates comparable to those not on TB therapy.^23^ Conversely, some observational studies have reported higher odds of virological failure among HIV/TB-coinfected individuals,^24^ highlighting the need to better understand factors that may modify HIV treatment response during concomitant TB/HIV therapy.

The dynamics between anti-TB drugs, ART, SNPs, and HIV-associated TB treatment outcomes are complex and not yet fully understood. This study described the SNPs of the Brazilian population and evaluated the relationship between SNPs known to be associated with ART metabolism and HIV virologic suppression among TB/HIV participants in a large, prospective, cohort study in Brazil. Additionally, we assessed the impact of other key determinants of HIV-1 virologic suppression, including baseline viral load (BVL), CD4 count, and timing of ART initiation.

## SUBJECTS AND METHODS

*Study design and population* - The Regional Prospective Observational Research in Tuberculosis (RePORT)-Brazil study enrolled participants with newly diagnosed, culture-confirmed, pulmonary TB at five sites across three regions in Brazil, between June 2015 and June 2019, and followed participants for two years after TB treatment initiation. Sites were in Rio de Janeiro (Instituto Nacional de Infectologia Evandro Chagas, Clínica de Saúde Rinaldo Delmare, Secretaria de Saúde de Duque de Caxias), Salvador (Instituto Brasileiro para Investigação da Tuberculose), and Manaus (Fundação Medicina Tropical Dr Heitor Vieira Dourado). The RePORT-Brazil population is broadly representative of TB cases in Brazil, as described previously.^25^ For this study, we included RePORT-Brazil participants with TB/HIV who initiated standard TB therapy and received ART during TB treatment.

*Variables and definitions* - The standard TB regimen followed Brazilian National TB Program guidelines, and was defined as a two-month intensive phase of isoniazid, rifampicin or rifabutin, pyrazinamide, and ethambutol, followed by a four-month (or more) continuation phase of isoniazid and rifampicin or rifabutin.^26^ TB treatment outcomes followed the World Health Organization (WHO) definition and were defined as favorable, a combination of cure and treatment completion, and as unfavorable, which comprised death, treatment failure, loss to follow up, and transferred.^27^

Clinical, demographic, and socio-economic data were collected longitudinally at baseline, month 2, and end of TB treatment visits, and during the two-year follow-up period; for the latter, participants were contacted by telephone to assess signs and symptoms of TB recurrence. Adherence in RePORT-Brazil included different modalities of directly observed therapy (DOT), such as in-person observation, phone calls, text messages, and video calls.

All participants underwent HIV testing at baseline unless they were already known to be a person with HIV/AIDS (PWH). We collected data on ART, ART timing initiation in relation to TB treatment, CD4 cell count, and HIV-1 RNA viral load (VL). We classified ART regimens according to the main antiretroviral class that composed the 3-drug regimen:^28^ non-nucleoside reverse transcriptase inhibitors (NNRTIs), protease inhibitors (PIs), and integrase strand transfer inhibitors (INSTIs). We considered only ART used during TB treatment and our focus was on NNRTI [efavirenz (EFZ)] and INSTI (dolutegravir, raltegravir), as these regimens were recommended ART in Brazil for TB/HIV coinfection during the study period.^28^ The dose of EFZ was 600 mg, administered once daily; raltegravir was administered at 400 mg twice daily; and dolutegravir was given at 50 mg twice daily, with the double dose due to a drug interaction with rifampicin. CD4 was categorized as: ≤ 200 cells/µL vs > 200 cells/µL. We considered VL as a continuous (copies/mL and Log_10_) and as a categorical variable and, for the primary analysis, we defined virologic suppression as ≤ 1,000 HIV-1 RNA viral copies/mL.^29^ For the secondary analysis, we considered virologic suppression as ≤ 50 HIV-1 RNA viral copies/mL.

RePORT-Brazil genotyped 60 selected polymorphisms in 29 genes relevant to TB or HIV drug metabolism, and for this study, we selected *CYP2B6* and *UGT1A1*, which are associated with the metabolism of EFZ and dolutegravir/raltegravir, respectively. Genotyping was done using MassARRAY® iPLEX Gold (Agena Bioscience™, California, USA) and Taqman (ThermoFisher Scientific, Massachusetts, USA).

*Statistical analysis* - Participant characteristics were described according to virologic suppression, summarizing continuous variables with median and interquartile range (IQR) and categorical variables with frequency and percentages.

Composite *CYP2B6* metabolizer genotype was defined based on combinations of three polymorphisms as follows: normal (1: 15582CC-516GG-983TT or 2: 15582CT-516GG-983TT); intermediate (3: 15582TT-516GG-983TT; 4: 15582CC-516GT-983TT; 5: 15582CC-516GG-983CT; 6: 15582CT-516GT-983TT; or 7: 15582CT-516GG-983CT); and slow (8: 15582CC-516TT-983TT; 9: 15582CC-516GT-983CT; 10: 15582CC-516GG-983CC).^30^ And the *UGT1A1* metabolizer genotype was defined as normal (887829CC), intermediate (887829CT), or slow (887829TT).^31^ The normal genotypes for *CYP2B6* and *UGT1A1* are the inducers of the enzymatic system, and thus, metabolize ARV faster.^12^ ^,19^

The endpoint was virologic suppression during TB treatment. Categorical variables were analyzed using the chi-square test, and continuous variables were assessed using the Mann-Whitney U test. Time-to-suppression was defined as the number of days from TB treatment initiation for ART-experienced participants or from ART initiation for ART-naïve participants until the end of TB treatment. Kaplan-Meier curves were constructed to analyze time-to-virologic suppression, overall and stratified by ART initiation time (before TB treatment vs ART naïve), BVL (< 5 log_10_ vs ≥ 5 log_10_), CD4 cell count (above vs below 200 cells/µL), and SNP categories (normal, intermediate, and slow). Kaplan-Meier curves were compared using the log-rank test. Univariable and multivariable Cox proportional hazards analyses were conducted to evaluate factors associated with the hazard of virologic suppression — multivariate analysis was adjusted for age, sex, site, ART initiation, baseline CD4 cell count, and BVL; and in the sub analysis, according to the *UGT1A1* and *CYP2B6* genotypes. All statistical analyses were performed using SPSS version 25.0 and RStudio v2026.01.0+392, with a significance level of 0.05.

*Ethics* - The RePORT-Brazil study was approved by the institutional review board of the Instituto Nacional de Infectologia Evandro Chagas (CAAE: 25102412.3.1001.5262), by the institutional review boards of the other study sites, and Vanderbilt University Medical Center. Written informed consent was obtained from all participants, and all clinical investigations were conducted according to the principles expressed in the Declaration of Helsinki.

## RESULTS

**Fig. 1:**
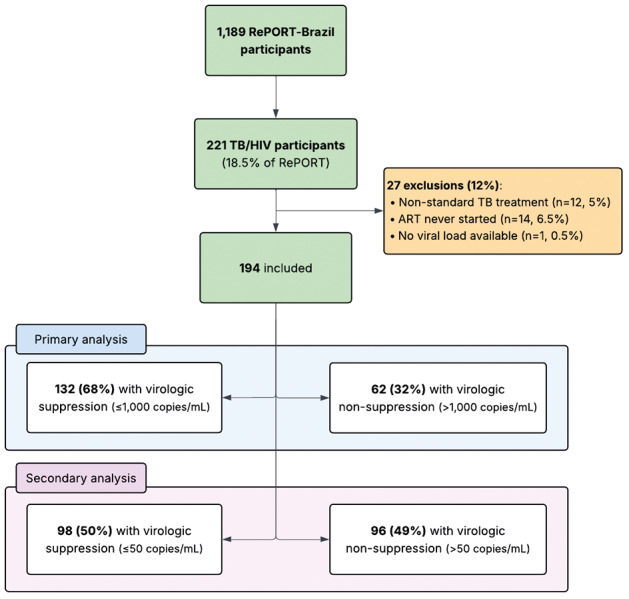
study diagram. RePORT: Regional Prospective Observational Research on Tuberculosis; TB: tuberculosis; ART: antiretroviral therapy.

Among 1,189 participants with TB in RePORT-Brazil, 221 (18.5%) were PWH, and 194 (88%) were included in the analysis (Fig. 1). Virologic suppression rates were low overall, with 132 participants (68%) achieving the primary endpoint (≤ 1,000 copies/mL). Compared to those who did not achieve suppression, participants with suppressed VL had higher median weight (55 kg vs 53 kg, p = 0.04), and lower log_10_ VL in baseline (3.8 vs 5.1, p < 0.001). They also had higher median CD4 cell counts at baseline (144 vs 84, p = 0.01) and at month 2 (145 vs 82, p = 0.02), higher rates of favorable TB treatment outcome (82% vs. 35%, p < 0.001) and lower rates of mortality (4% vs. 26%) and loss to follow-up (11% vs. 37%). Participant from Manaus site had higher proportions of non-suppressed VL compared to Rio de Janeiro and Salvador sites (p = 0.03). Overall, INSTI-based regimens were the most used ART [53% among participants with virologic suppression (n = 70) and 48% among non-suppressed (n = 30)]. NNRTI-based regimens were used in 37% of participants with virologic suppression (n = 49) and 45% of those without suppression (n = 28). Regarding pharmacogenetic profiles, *UGT1A1* normal metabolizer genotype and *CYP2B6* intermediate metabolizer genotype were the most prevalent in the study population, irrespectively of virologic suppression (Table I). Secondary analysis for the endpoint of ≤ 50 copies/mL revealed similar results and associations [Supplementary data (Table I)].

**TABLE I:** Study population characteristics (N = 194), stratified by human immunodeficiency virus (HIV)-1 virologic suppression (≤ 1000 copies/mL vs. >1000 copies/mL)

	Suppressed (≤ 1000 copies/mL) N = 132, 68%		Non-suppressed (> 1000 copies/mL) N = 62, 32%		p-value^1^
	Median or N (IQR or %)				
Age	35	(28-41)	36	(28-44)	0.58
Sex					
Male	103	(78)	47	(76)	0.73
Weight	55	49-66	53	48-60	**0.05**
Site (city)					
Rio de Janeiro	40	(30)	8	(13)	**0.02**
Manaus	91	(69)	52	(84)	
Salvador	1	(1)	2	(3)	
Race					
White	26	(20)	8	(13)	0.46
Black	21	(16)	7	(11)	
Brown	82	(62)	45	(73)	
Other	3	(2)	2	(3)	
Educational level (Literate)	128	(97)	61	(98)	0.56
Years of education	10	(6-12)	8	(5-11)	0.08
Household income^2^					
> 1 min wage	39	(30)	22	(35)	0.87
≤ 1 min wage	49	(37)	21	(34)	
Unknown	3	(2)	1	(2)	
No reported	40	(31)	18	(29)	
BCG scar					
yes	106	(80)	44	(71)	0.15
Previous TB					
yes	27	(20)	9	(15)	0.54
HIV-1 viral load (copies/mL and log_10_)					
Baseline	6,541	(90-117,585)	133,977	(31,549-334,145)	**<0.001**
log_10_	3.8	(2-5.1)	5.1	(4.5-5.5)	
High (≥ 5 log_10_ [~100,000 copies/mL])	36	(28)	35	(60)	
Low (< 5 log_10_)	93	(72)	24	(40)	
CD4 count (cells/µL)					
Baseline	144	(56-316)	84	(43-175)	**0.03**
≤ 200	82	(63)	48	(79)	
> 200	48	(37)	13	(21)	
Month 2	145	(71-301)	82	(58-177)	**0.01**
≤ 200	73	(60)	33	(83)	
> 200	48	(40)	7	(17)	
End of TB	281	(159-411)	188	(93-329)	0.06
≤ 200	43	(40)	10	(50)	0.14
> 200	60	(60)	10	(50)	
ART initiation					
Before TB treatment	62	(47)	21	(34)	0.09
ART naïve	70	(53)	41	(66)	
Main ART regimen					
PI	12	(9)	4	(6)	0.68
NNRTI	49	(37)	28	(45)	
INSTI	70	(53)	30	(48)	
PI+INSTI	1	(1)	--	--	
Main ART drug					
Efavirenz	48	(36)	28	(45)	0.44
Dolutegravir	7	(5)	5	(8)	
Raltegravir	63	(48)	25	(40)	
Atazanavir	8	(6)	3	(5)	
Lopinavir	--	--	1	(2)	
Darunavir	4	(3)	--	--	
Darunavir + Raltegravir	1	(1)	--	--	
Etravirine	1	(1)	--	--	
*CYP2B6* Metabolizer profiles					
Normal	29	(24)	17	(35)	0.26
Intermediate	74	(60)	23	(48)	
Slow	20	(16)	8	(17)	
*UGT1A1* Metabolizer profiles					
Normal	60	(49)	22	(48)	0.09
Intermediate	49	(40)	13	(28)	
Slow	14	(11)	11	(24)	
TB treatment outcomes					
Favorable	108	(82)	22	(35)	**<0.001**
Unfavorable	24	(18)	40	(65)	
TB treatment outcomes (description)					
Cure	65	(49)	17	(27)	**<0.001**
Treatment complete	43	(33)	5	(8)	
Death	5	(4)	16	(26)	
Treatment failure	5	(4)	--	--	
LTFU	14	(11)	23	(37)	
Transferred	--	--	1	(2)	

ART: antiretroviral therapy; PI: protease inhibitor; NNRTI: non-nucleoside reverse transcriptase inhibitors: efavirenz; INSTI: integrase strand transfer inhibitors; LTFU: loss to follow-up; TB: tuberculosis. ^1^Statistical tests: categorical variables Chi squared test, continuous variables: Mann-Whitney test, significance level .05. ^2^The Brazilian minimum wage is approximately USD 286.00 per month.

**Fig. 2:**
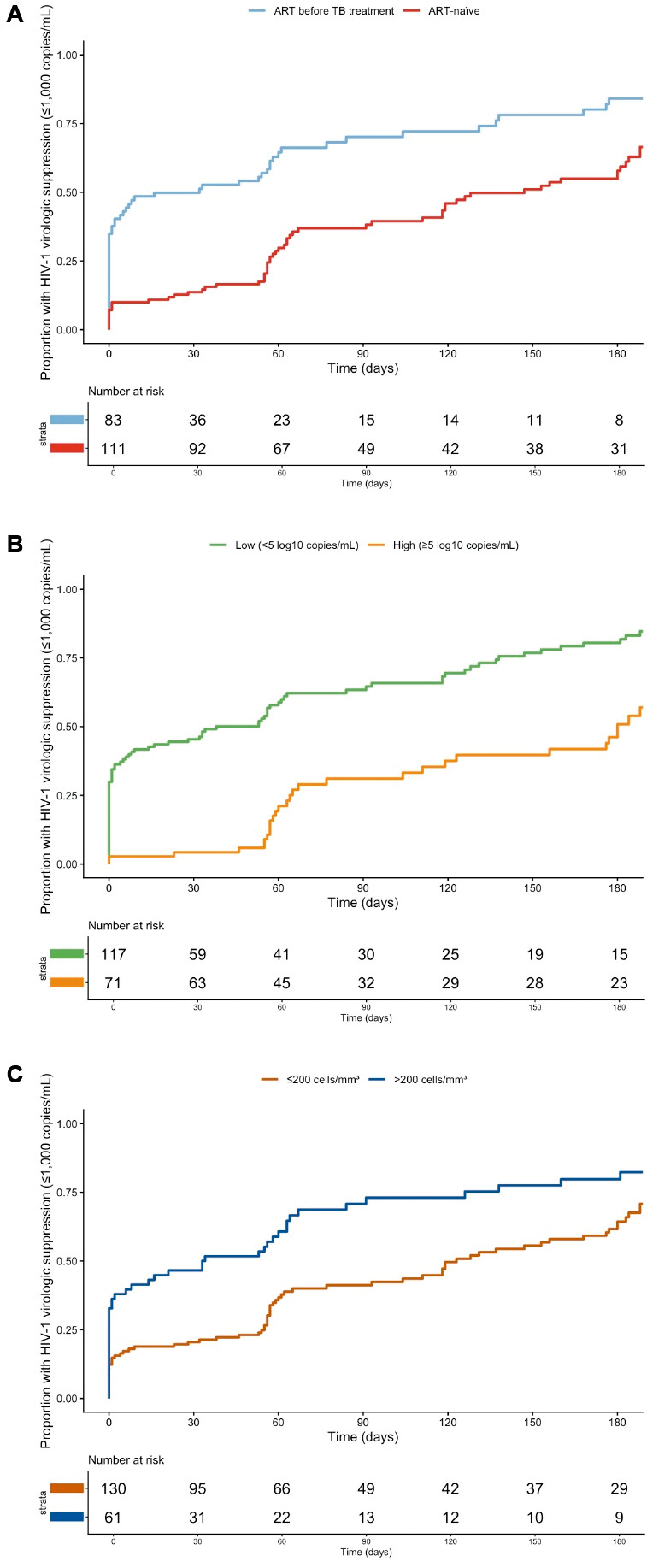
Kaplan-Meier curves of time-to-virologic suppression (≤ 1,000 copies/mL) according to (A) antiretroviral therapy (ART) initiation, (B) baseline viral load (BVL), and (C) baseline CD4 cell count, among all participants (N = 194). Numbers at risk are shown below the graph. (A) Virologic suppression occurred earlier in participants who initiated ART before tuberculosis (TB) treatment [median: six days; 95% confidence interval (CI): 0-53] compared to ART-naïve participants (median: 64 days; 95% CI: 60-118), log-rank χ² = 15.1, p < 0.001. (B) Suppression was also quicker in participants with low BVL (< 5 log_10_; median: 30 days; 95% CI: 5-56) compared to those with high BVL (≥ 5 log_10_ [~100,000 copies]; median: 67 days; 95% CI: 60-176), log-rank χ² = 12.6, p < 0.001. (C) Participants with baseline CD4 counts > 200 cells/µL achieved suppression faster (median: 33 days; 95% CI: 1-60) than those with ≤ 200 cells/µL (median: 60 days; 95% CI: 56-67), log-rank χ² = 5.02, p = 0.02.

The median time-to-virologic suppression, irrespective of ART regimen, was 84 days (IQR: 42–125) for the primary endpoint (≤ 1,000 copies/mL) and 194 days (IQR 174-213) for the secondary endpoint (≤ 50 copies/mL). In primary analysis, participants who initiated ART before TB treatment achieved virologic suppression significantly faster compared to ART-naïve individuals (median days: 6 vs 64 days, log-rank χ² = 48.5, p < 0.001). Similarly, participants with lower BVL (< 5 log_10_) and higher CD4 counts (> 200 cells/µL) had shorter median time-to-suppression compared to those with higher VL (≥ 5 log_10_) and lower CD4 counts (≤ 200 cells/µL), log-rank χ² = 12.6 and 5.02, respectively, with p < 0.001 and p = 0.02, respectively (Fig. 2). Secondary analysis showed comparable trends [Supplementary data (Fig. 1)].

**Fig. 3:**
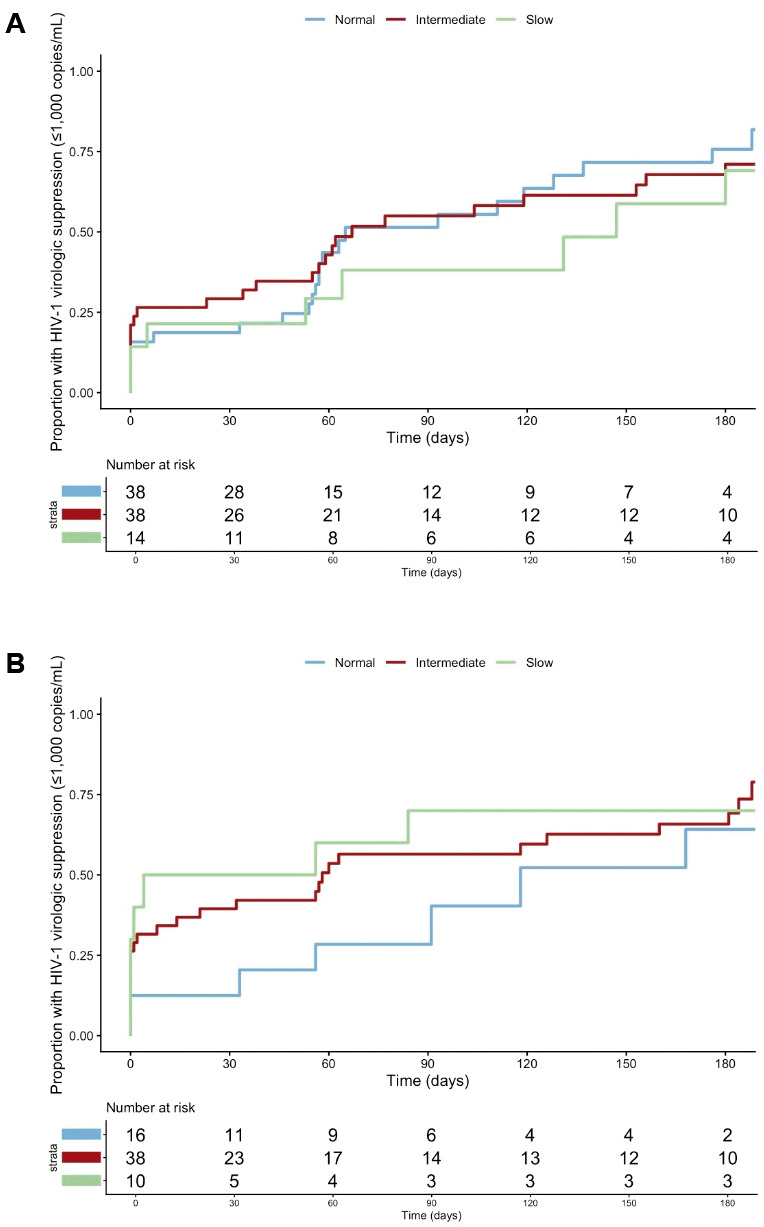
Kaplan-Meier curves of time-to-virologic suppression (≤ 1,000 copies/mL) according to (A) *UGT1A1* genotypes among participants on integrase strand transfer inhibitors (INSTI)-based regimens and (B) *CYP2B6* genotypes among participants on efavirenz (EFZ)-based regimens. Numbers at risk are shown below each graph. (A) For INSTI-based regimens (N = 90), median time to virologic suppression was 57 days [95% confidence interval (CI): 54-93] for normal *UGT1A1* genotypes, 62 days (95% CI: 38-153) for intermediate genotypes, and 75 days (95% CI: 53-196) for slow genotypes, with no statistically significant differences (log-rank test χ² = 2.53, p = 0.283). (B) For EFZ-based regimens (N = 64), median time to virologic suppression was 60 days (95% CI: 27-177) for normal *CYP2B6* genotypes, 57 days (95% CI: 14-160) for intermediate genotypes, and 30 days (95% CI: 0-89) for slow genotypes, with no statistically significant differences (log-rank test: χ² = 1.5, p = 0.48). Note: among participants on INSTI-based regimens (N = 100), *UGT1A1* genotyping was performed for 90% (N = 90). Similarly, *CYP2B6* genotyping was performed for 84% (N = 64) of participants on EFZ-based regimens (N = 76).

Among participants receiving an INSTI-based regimen (N = 100), the median time-to-virologic suppression was 57 days (IQR: 54-93) for participants with *UGT1A1* normal genotypes, 62 days (IQR: 38-153) for those with intermediate genotypes, and 75 days (IQR: 53-196) for those with slow genotypes (log-rank χ² = 2.53, p = 0.283) (Fig. 3A). Similarly, among participants receiving an EFZ-based regimen (N = 76), the median time-to-virologic suppression was 60 days (IQR: 27-177) for participants with *CYP2B6* normal genotypes, 57 days (IQR: 14-160) for those with intermediate genotypes, and 30 days (IQR: 0-89) for those with slow genotypes (log-rank χ² = 1.5, p = 0.48) (Fig. 3B). For the secondary endpoint (≤ 50 copies/mL), we observed longer time-to-virologic suppression for all genotypes, but with no statistical difference among them [Supplementary data (Fig. 2)].

In univariable Cox regression, a lower hazard of suppression was associated with higher BVL [hazard ratio (HR): 0.65, 95% confidence interval (CI): 0.59-0.71, p < 0.001] and ART-naïve status (HR: 0.49, CI: 0.34-0.69, p < 0.001). A higher hazard of suppression was associated with increased baseline CD4 count (per 100 cell/µL, HR: 1.22, 95% CI: 1.12-1.31, p < 0.001), as well as being from Rio de Janeiro site, compared to Manaus site (HR: 1.49, 95% CI: 1.02-2.17). Age, sex, and race were not statistically significant predictors at the 5% level. In multivariable analysis, baseline CD4 count [per 100 cell/µL increase, adjusted hazard ratio (aHR): 1.10, 95% CI: 1.02-1.20, p = 0.02] and BVL (aHR: 0.76, 95% CI: 0.69-0.83, p < 0.001) remained statistically significant at the 5% level, while timing of ART initiation, age, sex, and site were not statistically significant (Table II). Similar findings were observed for the secondary endpoint (≤ 50 copies/mL) [Supplementary data (Table II)].

**TABLE II:** Univariate and multivariate Cox regression analysis of predictors of human immunodeficiency virus (HIV)-1 virologic suppression (≤ 1000 copies/mL) among all study participants (N = 194)

	Univariate analysis		Multivariate analysis	
Variable	HR (95% CI)	p-value	aHR (95% CI)	p-value
Age (per year increase)	1.00 (0.99-1.02)	0.62	1.00 (0.99-1.02)	0.84
Sex				
Male	**Ref.**	—	**Ref.**	—
Female	0.84 (0.55-1.27)	0.41	0.81 (0.57-1.15)	0.24
Site				
Manaus	**Ref.**	—	**Ref.**	—
Rio de Janeiro	**1.49 (1.02-2.17)**	**0.04**	0.79 (0.55-1.15)	0.22
Salvador	0.51 (0.07-3.69)	0.51	0.74 (0.22-2.47)	0.63
Baseline CD4 count (per 100 cells/µL)	**1.22 (1.12-1.31)**	**<0.001**	**1.10 (1.02-1.20)**	**0.02**
Baseline HIV-1 viral load (log_10_)	**0.65 (0.59-0.71)**	**<0.001**	**0.76 (0.69-0.83)**	**<0.001**
ART status				
Before TB treatment	**Ref.**	—		
ART naïve	**0.49 (0.34-0.69)**	**<0.001**	0.74 (0.54-1.02)	0.07
Race				
White	**Ref.**	—	—	—
Black	0.88 (0.49-1.59)	0.666	—	—
Brown	0.75 (0.48-1.14)	0.735	—	—
Other	0.81 (0.24-2.68)	0.726	—	—
*UGT1A1* genotypes				
Normal	**Ref.**	—	—	—
Intermediate	0.90 (0.52-1.56)	0.71	—	—
Slow	0.75 (0.35-1.62)	0.47	—	—
*CYP2B6* genotypes				
Normal	**Ref.**	—	—	—
Intermediate	1.60 (0.73-3.51)	0.24	—	—
Slow	1.66 (0.62-4.49)	0.32	—	—

ART: antiretroviral therapy; HR: hazard ratio; aHR: adjusted hazard ratio; 95% CI: 95% confidence interval. Significance level .05

Subgroup analyses were conducted for participants receiving INSTI- or EFZ-based ART regimens (Table III). In multivariate analysis, among INSTI-based regimen users, baseline CD4 count (per 100 cell/µL increase, aHR: 1.17, 95% CI: 1.03-1.34, p = 0.01) and BVL (aHR: 0.78, 95% CI: 0.70-0.88, p < 0.001) were significantly associated with virologic suppression. *UGT1A1* genotypes were not associated with virologic suppression in either univariate or multivariate analyses, as intermediate metabolizers (p = 0.43) and slow metabolizers (p = 0.39) showed no significant differences compared to normal metabolizers. Age, sex, and site were also not significant predictors, either in uni- or multivariate analysis. For EFZ-based regimen users, BVL (aHR: 0.58, 95% CI: 0.47-0.72, p < 0.001) remained a significant predictor of suppression, while baseline CD4 count was significant only in univariate analysis (HR: 1.24, 95% CI: 1.09-1.40, p < 0.001). *CYP2B6* genotype was not significantly associated with virologic suppression in either univariate or multivariate analyses, with intermediate metabolizers (p = 0.68) and slow metabolizers (p = 0.51) showing no differences compared to normal metabolizers. Age, sex, and site were also not significant predictors in the EFZ group. The secondary endpoint analysis demonstrated similar results, except for *CYP2B6* genotypes — in the multivariate analysis, *CYP2B6* intermediate metabolizers were significantly associated with a higher likelihood of virologic suppression compared to normal metabolizers (aHR: 0.59, 95% CI: 0.36-0.95, p = 0.03) [Supplementary data (Table III)].

**TABLE III:** Univariate and multivariate Cox regression analysis of predictors of human immunodeficiency virus (HIV)-1 virologic suppression (≤ 1000 copies/mL) among participants with integrase strand transfer inhibitors (INSTI)- and efavirenz (EFZ)-based antiretroviral therapy (ART) regimens

	Univariate analysis		Multivariate analysis	
Variable	HR (95% CI)	p-value	aHR (95% CI)	p-value
**INSTI (N = 100)**				
Age (per year increase)	0.99 (0.97-1.02)	0.66	0.99 (0.96-1.01)	0.33
Sex				
Male	**Ref.**	—	**Ref.**	—
Female	0.89 (0.50-1.60)	0.71	0.63 (0.36-1.09)	0.10
Site				
Manaus	**Ref.**	—	**Ref.**	—
Rio de Janeiro	1.67 (0.94-2.97)	0.08	1.32 (0.72-2.40)	0.37
Salvador	0.00 (0.00-Inf)	1.00	1.21 (0.14-10.17)	0.86
Baseline CD4 count (per 100 cells/µL)	**1.18 (1.04-1.34)**	**0.01**	**1.17 (1.03-1.34)**	**0.01**
Baseline HIV-1 viral load (log_10_)	**0.71 (0.63-0.81)**	**<0.001**	**0.78 (0.70-0.88)**	**<0.001**
*UGT1A1* genotypes				
Normal	**Ref.**	—	**Ref.**	—
Intermediate	0.90 (0.52-1.56)	0.71	0.82 (0.50-1.35)	0.43
Slow	0.75 (0.35-1.62)	0.47	0.75 (0.38-1.45)	0.39
**EFZ (N = 76)**				
Age (per year increase)	1.02 (0.99-1.04)	0.24	1.01 (0.99-1.03)	0.41
Sex				
Male	**Ref.**	—	**Ref.**	—
Female	0.57 (0.25-1.28)	0.17	0.87 (0.41-1.84)	0.72
Site				
Manaus	**Ref.**	—	**Ref.**	—
Rio de Janeiro	1.51 (0.78-2.91)	0.22	0.56 (0.26-1.19)	0.13
Salvador	3.94 (0.52-30.05)	0.19	0.69 (0.07-7.19)	0.75
Baseline CD4 count (per 100 cells/µL)	**1.24 (1.09-1.40)**	**<0.001**	1.04 (0.89-1.20)	0.64
Baseline HIV-1 viral load (log_10_)	**0.58 (0.49-0.69)**	**<0.001**	**0.62 (0.51-0.76)**	**<0.001**
*CYP2B6* genotypes				
Normal	**Ref.**	—	**Ref.**	—
Intermediate	1.60 (0.73-3.51)	0.24	1.15 (0.58-2.29)	0.68
Slow	1.66 (0.62-4.49)	0.32	0.72 (0.27-1.90)	0.51

HR: hazard ratio; aHR: adjusted hazard ratio; 95% CI: 95% confidence interval. Significance level .05; Note: among participants on INSTI-based regimens (N = 100), *UGT1A1* genotyping was performed for 90% (N = 90). Similarly, *CYP2B6* genotyping was performed for 84% (N = 64) of participants on EFZ-based regimens (N = 76).

## DISCUSSION

In this prospective cohort of participants with HIV-associated TB, the virologic suppression was suboptimal, with approximately two-thirds reaching the primary endpoint (≤ 1,000 copies/mL), and fewer than half reaching the secondary endpoint (≤ 50 copies/mL). The HIV disease severity markers, such as BVL and CD4 cell count, were the main determinants of virologic suppression in this setting.

Participants who entered TB care with lower VL and higher CD4 counts had substantially faster and more frequent virologic suppression, associations which persisted in multivariable models and across sensitivity analyses. This pattern aligns with established HIV treatment, with additional relevance in TB/HIV patients, who often present with advanced immunosuppression and face barriers to rapid virologic control, such as pill burden, inflammation, and drug-drug interactions.^21^ ^,23^ ^,32^ ^33^,^34^,^35^,^36^,^37^

Although participants from the Rio de Janeiro site showed higher hazard of virologic suppression compared with Manaus in univariate analyses, this association did not persist after multivariable adjustment, indicating that site-related differences were explained by underlying clinical and epidemiological characteristics rather than a site-specific effect. Manaus site is a tertiary HIV referral center recruiting participants directly from routine clinical care, often during hospitalization, which likely results in the inclusion of individuals with more advanced disease and greater clinical complexity. In addition, the epidemiology of HIV and TB in the Brazilian Amazon, where Manaus is located, is characterized by higher mortality rates and increased TB and HIV incidences, which may further contribute to the more vulnerable clinical profile observed at this site.^38^ ^,39^

INSTI-based regimens were more commonly used than EFZ-based regimens. This may be explained by a trend in recommendation for primary ART regimen for treatment-naïve TB/HIV patients in Brazil in 2017. Moreover, rates of resistance to EFZ in the world and in Brazil is high — ranging from 3.4% to 5.5% and could exceed 10%.^40^ This precludes using EFZ-based regimens as a first-line ART without baseline HIV-1 genotyping.^41^ ^,42^ For that reason, since 2017, for TB/HIV patients meeting the criteria of severe disease — *i.e.*, CD4 cell count < 100 cells/µL, disseminated TB, other concomitant opportunistic infection, and hospitalized patients —, the recommended ART regimen was to include raltegravir; and EFZ-based treatment was recommended for TB/HIV without severe disease. However, since 2019, the first-line ART regimen recommended for TB/HIV participants has been a double dose dolutegravir-based ART regimen, expecting to increase effectiveness as dolutegravir is a safe and well tolerated drug, with a higher genetic barrier to resistance.^43^ Raltegravir is no longer recommended as an option for TB/HIV patients.^8^ ^,44^ EFZ-based regimens are also well-tolerated, but neuropsychiatric adverse reactions, and primary and acquired resistance, can limit its use.^45^

Despite these differences in regimen availability and guideline evolution, ART regimen class did not meaningfully influence virologic outcomes in our study. Participants who were already on ART before TB treatment suppressed markedly faster than ART-naïve participants, demonstrating that initiating ART during TB treatment can be challenging.^46^ ^,47^ ^,48^ ^,49^ Different from the literature,^50^ ^,51^ participants receiving INSTI-based regimens had virologic suppression rates and times comparable to those on NNRTI-based therapy. This is notable because INSTIs, especially dolutegravir, are known to achieve rapid viral decay.^20^ ^,46^ ^,47^ ^,50^ Moreover, participants included in this study were previously enrolled in RePORT-Brazil study, which has different DOT modalities.

The pharmacogenetic profiles — *UGT1A1* metabolizer status for INSTI users and *CYP2B6* metabolizer status for EFZ users — were not associated with virologic suppression in primary analyses. Although these genetic variants influence antiretroviral drug exposure,^18^ ^,19^ ^,20^ ^,30^ they did not impact the outcomes in this population.

Our study had limitations. We were unable to handle the VL as a continuous variable to the lack of consistent measurement across participants at the same time points during follow-up, as this was an observational, clinical practice-based study. The sample size for genotype-specific subgroup analyses was modest, potentially underpowering detection of associations. In addition, we did not assess ART toxicity or ART drug levels, which can contribute to virologic suppression. Conversely, we highlight that we had a prospective cohort study that is representative of the Brazilian population,^25^ with important data on human genetic variants and ART, as well as treatment outcomes, and *UGT1A1* and *CYP2B6* metabolizer profiles.

In this observational cohort of patients treated for TB/HIV, baseline HIV disease severity markers — BVL and CD4 count — were the main determinants of virologic suppression during TB treatment. The overall proportion of participants achieving virologic suppression was low, and neither ART regimens nor their associated pharmacogenetic variants were significantly associated with the likelihood of suppression.

## SUPPLEMENTARY MATERIALS

Supplementary data

## Data Availability

The datasets used and analyzed during the current study are available from the corresponding author on reasonable request.
