# Supplementary material for: Determinants of HIV-1 virologic suppression in HIV-associated tuberculosis in Brazil
Source: Mem Inst Oswaldo Cruz. 2026 Jul 10;121:e250090. doi: 10.1590/0074-02760250090 (PMC13336628; doi:10.1590/0074-02760250090)
Supplement: Supplementary data [file 1678-8060-mioc-121-e250090-s1.pdf]

TABLE I

Study population characteristics (N = 194), stratified by human immunodeficiency virus-1 virologic suppression (≤ 50 copies/mL vs. > 50 copies/mL)

	Suppressed (≤ 50 copies/mL) N = 98, 50%		Non-suppressed (> 50 copies/mL) N = 96, 49%		
	Median or N (IQR or %)				p-value[1]
Age	36	(28-43)	35	(28-42)	0.28
Sex					
Male	77	(79)	73	(76)	0.67
Weight	55	(47-66)	54	(49-62)	0.50
Site (city)					
Rio de Janeiro	32	(33)	16	(17)	**0.03**
Manaus	65	(66)	78	(81)	
Salvador	1	(1)	2	(2)	
Race					
White	18	(18)	16	(17)	0.35
Black	17	(17)	11	(12)	
Brown	62	(63)	65	(68)	
Other	1	(1)	4	(4)	
Educational level (Literate)	94	(96)	95	(99)	0.18
Years of education	9	6-12	10	5-12	0.92
Household income[2]					
> 1 min wage	30	(31)	31	(32)	0.67
≤ 1 min wage	39	(40)	31	(32)	
Uknown	2	(2)	2	(2)	
No reported	26	(27)	32	(33)	
BCG scar					
yes	78	(80)	72	(75)	0.45
Previous TB					
yes	20	(20)	16	(17)	0.80
HIV-1 viral load (copies/mL and $\log_{10}$)					
Baseline	9,235	(0-104,241)	83,820	(2,372-334,145)	**<0.001**
$\log_{10}$	3.9	(0-5)	4.9	(3.4-5.5)	**<0.001**
High (≥ 5 $\log_{10}$ [~100,000 copies/mL])	25	(26)	46	(49)	**0.001**
Low (< 5 $\log_{10}$)	70	(74)	47	(51)	
CD4 count (cells/µL)					
Baseline	150	(57-316)	90	(43-196)	**0.04**
≤ 200	59	(60)	71	(76)	**0.03**
> 200	38	(40)	23	(24)	
Month 2	144	(66-305)	98	(68-185)	**0.05**
≤ 200	54	(59)	52	(75)	0.03
> 200	38	(41)	17	(25)	
End of TB	284	(172-418)	205	(111-331)	**0.02**
≤ 200	32	(38)	21	(49)	0.24
> 200	50	(62)	22	(51)	

	Suppressed (≤ 50 copies/mL) N = 98, 50%		Non-suppressed (> 50 copies/mL) N = 96, 49%		
	Median or N (IQR or %)				p-value[1]
ART initiation					
Before TB treatment	41	(42)	42	(44)	0.79
ART naïve	57	(58)	54	(56)	
Main ART regimen					
PI	7	(7)	9	(9)	0.69
NNRTI	39	(40)	38	(40)	
INSTI	51	(52)	49	(51)	
PI + INSTI	1	(1)	--	--	
Main ART drug					
Efavirenz	38	(39)	38	(40)	0.68
Dolutegravir	4	(4)	8	(8)	
Raltegravir	47	(48)	41	(43)	
Atazanavir	5	(5)	6	(6)	
Lopinavir	--	--	1	(1)	
Darunavir	2	(2)	2	(2)	
Darunavir + Raltegravir	1	(1)	--	--	
Etravirine	1	(1)	--	--	
CYP2B6 Metabolizer profiles					
Normal	25	(31)	21	(23)	0.43
Intermediate	45	(56)	52	(58)	
Slow	11	(14)	17	(19)	
UGT1A1 Metabolizer profiles					
Normal	37	(47)	45	(50)	0.60
Intermediate	28	(35)	34	(38)	
Slow	14	(18)	11	(12)	
TB treatment outcomes					
Favorable	84	(86)	46	(48)	**<0.001**
Unfavorable	14	(14)	50	(52)	
TB treatment outcomes (description)					
Cure	49	(50)	33	(34)	**<0.001**
Treatment complete	35	(36)	13	(14)	
Death	1	(1)	20	(21)	
Treatment failure	4	(4)	1	(1)	
LTFU	9	(9)	28	(29)	
Transferred	--	--	1	(1)	

ART: antiretroviral therapy; PI: protease inhibitor; NNRTI: non-nucleoside reverse transcriptase inhibitors: efavirenz; INSTI: integrase strand transfer inhibitors; LTFU: loss to follow-up. [1]Statistical tests. categorical variables: Chi squared test; continuous variables: Mann-Whitney test; significance level .05; [2]The Brazilian minimum wage is approximately USD 286.00 per month.

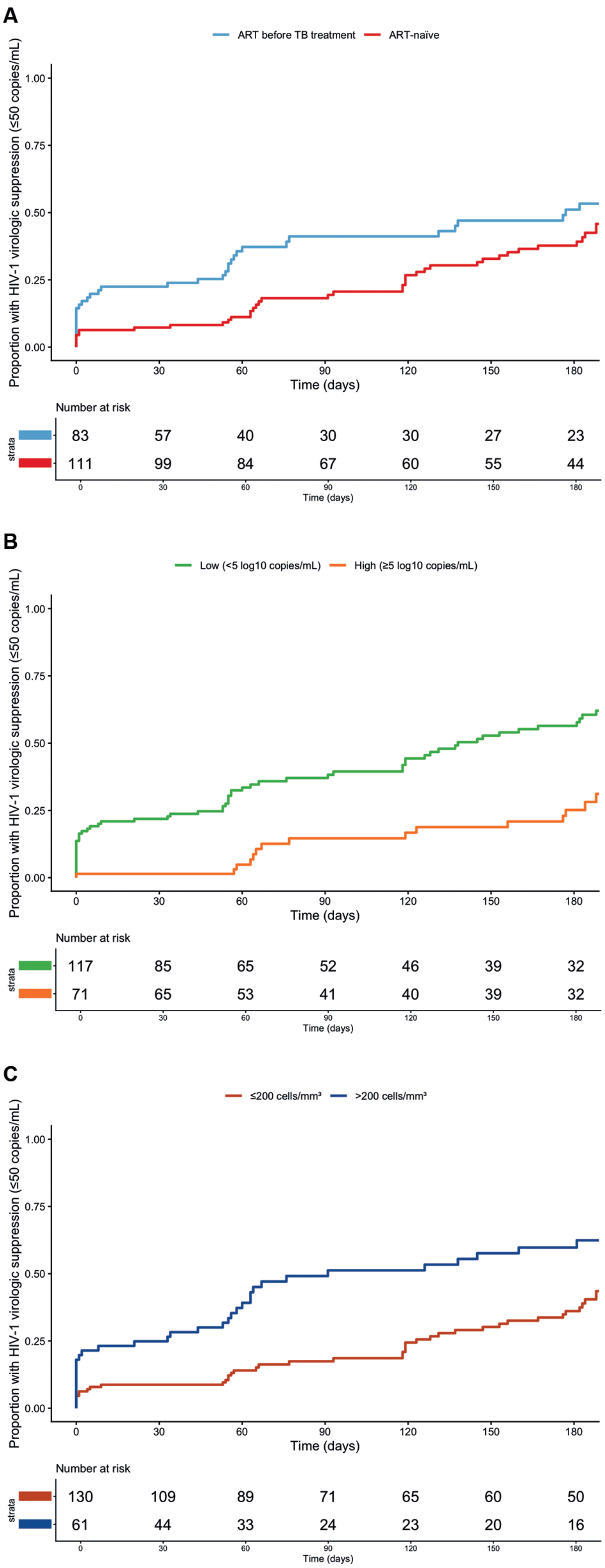

Fig. 1: Kaplan-Meier curves of time-to-virologic suppression (≤ 50 copies/mL) according to antiretroviral therapy (ART) initiation (A), baseline viral load (BVL) (B), and baseline CD4 cell count (C), among all participants (N = 194). Numbers at risk are shown below the graph. (A) Virologic suppression occurred in a median of 58 days [interquartile range (IQR) 55-76] for participants who initiated ART before tuberculosis (TB) treatment, compared to a median of 147 days (IQR 118-178) for ART-naïve participants (log-rank $\chi^2 = 6.46$, p = 0.011). (B) Suppression was also quicker in participants with low BVL (< 5 log_{10}; median: 63 days; IQR 56-119) compared to those with high BVL (≥ 5 log_{10} [~100,000 copies]; median: 335 days; IQR 77-182), log-rank $\chi^2 = 4.89$, p = 0.027.(C) Participants with baseline CD4 counts > 200 cells/µL achieved suppression faster (median: 63 days; IQR 55-138) than those with ≤ 200 cells/µL (median: 121 days; IQR 66-177), log-rank $\chi^2 = 5.62$, p = 0.018.

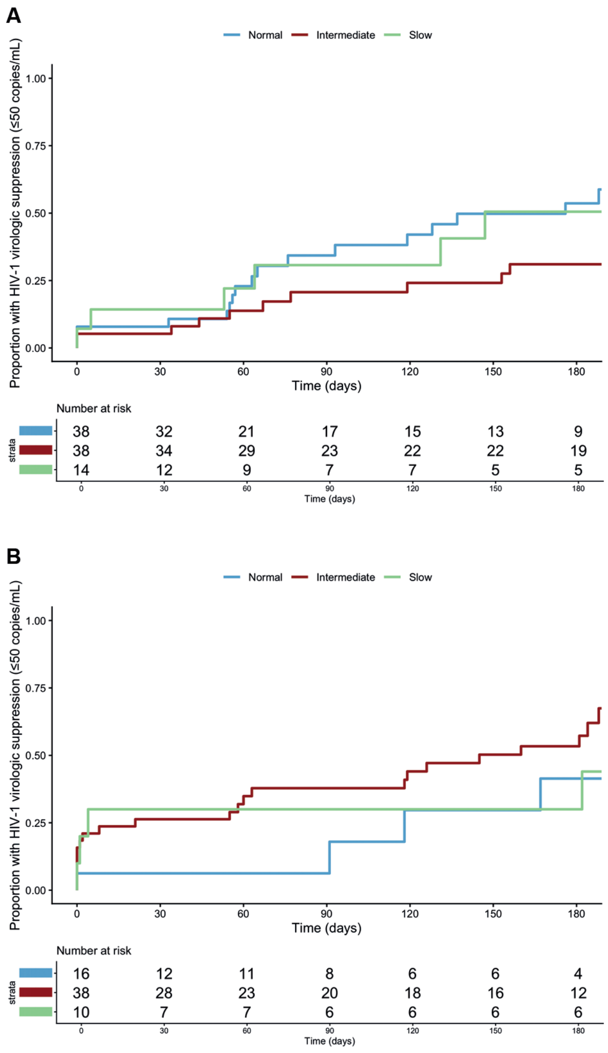

Fig. 2: Kaplan-Meier curves of time-to-virologic suppression (≤ 50 copies/mL) according to (A) *UGT1A1* genotypes among participants on integrase strand transfer inhibitors (INSTI)-based regimens and (B) *CYP2B6* genotypes among participants on efavirenz (EFZ)-based regimens. (A) For INSTI-based regimens (N = 90), median time-to-virologic suppression was 64 days [Interquartile range (IQR) 57-176] for normal *UGT1A1* genotypes, 178 days (IQR 67-196) for intermediate genotypes, and 109 days (IQR 54-196) for slow genotypes, with no statistically significant differences observed (log-rank test $\chi^2 = 3.56$, $p = 0.169$). (B) For EFZ-based regimens (N = 64), median time to virologic suppression was 78 days (IQR 31-183) for normal *CYP2B6* genotypes, 119 days (IQR 56-181) for intermediate genotypes, and 181 days (IQR 154-209) for slow genotypes, with no statistically significant differences observed (log-rank test: $\chi^2 = 1.47$, $p = 0.480$). Note: among participants on INSTI-based regimens (N = 100), *UGT1A1* genotyping was performed for 90% (N = 90). Similarly, *CYP2B6* genotyping was performed for 84% (N = 64) of participants on EFZ-based regimens (N = 76).

TABLE II

Univariate and multivariate Cox regression analysis of predictors of human immunodeficiency virus (HIV)-1 virologic suppression (≤ 50 copies/mL) among all study participants (N = 194)

	Univariate analysis		Multivariate analysis	
Variable	HR (95% CI)	p-value	aHR (95% CI)	p-value
Age (per year increase)	1.01 (1.00-1.03)	0.13	1.01 (0.99-1.02)	0.49
Sex				
Male	**Ref.**	—	**Ref.**	—
Female	0.80 (0.49-1.30)	0.38	0.86 (0.61-1.22)	0.40
Site				
Manaus	**Ref.**	—	**Ref.**	—
Rio de Janeiro	1.32 (0.85-2.05)	0.22	0.70 (0.48-1.02)	0.06
Salvador	1.05 (0.15-7.61)	0.96	1.30 (0.39-4.29)	0.67
Baseline CD4 count (per 100 cells/µL)	**1.26 (1.15-1.38)**	**<0.001**	1.09 (1.00-1.19)	0.06
Baseline HIV-1 viral load (log_{10})	**0.65 (0.58-0.73)**	**<0.001**	**0.82 (0.74-0.91)**	**<0.001**
ART statusBefore				
TB treatment	**Ref.**	—		
ART naïve	0.73 (0.48-1.09)	0.13	0.95 (0.69-1.32)	0.78
Race				
White	**Ref.**	—	—	—
Black	0.96 (0.47 - 1.93)	0.90	—	—
Brown	0.96 (0.57 - 1.63)	0.88	—	—
Other	0.48 (0.06 - 3.63)	0.48	—	—
UGT1A1 genotypes				
Normal	**Ref.**	—	—	—
Intermediate	0.63 (0.32-1.21)	0.16	—	—
Slow	1.24 (0.54-2.85)	0.62	—	—
CYP2B6 genotypes				
Normal	**Ref.**	—	—	—
Intermediate	2.25 (0.85-5.98)	0.10	—	—
Slow	1.46 (0.44-4.89)	0.54	—	—

ART: antiretroviral therapy; HR: hazard ratio; aHR: adjusted hazard ratio; 95% CI: 95% confidence interval. Significance level.

TABLE III

Univariate and multivariate Cox regression analysis of predictors of human immunodeficiency virus (HIV)-1 virologic suppression (≤ 50 copies/mL) among participants with: integrase strand transfer inhibitors (INSTI)- and efavirenz (EFZ)-based ART regimens

	Univariate analysis		Multivariate analysis	
Variable	HR (95% CI)	p-value	aHR (95% CI)	p-value
INSTI (N = 100)				
Age (per year increase)	1.00 (0.97-1.03)	0.96	0.98 (0.96-1.01)	0.22
Sex				
Male	**Ref.**	—	**Ref.**	—
Female	0.86 (0.42-1.80)	0.70	1.09 (0.64-1.87)	0.75
Site				
Manaus	**Ref.**	—	**Ref.**	—
Rio de Janeiro	**2.12 (1.09-4.12)**	**0.03**	1.42 (0.74-2.72)	0.29
Salvador	0.00 (0.00-Inf)	1.00	1.10 (0.13-9.41)	0.93
Baseline CD4 count (per 100 cells/μL)	**1.25 (1.07-1.45)**	**0.00**	1.12 (0.98-1.27)	0.11
Baseline HIV-1 viral load (log_{10})	**0.69 (0.58-0.81)**	**<0.001**	**0.84 (0.74-0.95)**	**0.00**
UGT1A1 genotypes				
Normal	**Ref.**	—	**Ref.**	—
Intermediate	0.63 (0.32-1.21)	0.16	**0.59 (0.36-0.95)**	**0.03**
Slow	1.24 (0.54-2.85)	0.62	0.89 (0.45-1.76)	0.74
EFZ (N = 76)				
Age (per year increase)	1.03 (1.00-1.06)	0.06	1.00 (0.98-1.03)	0.81
Sex				
Male	**Ref.**	—	**Ref.**	—
Female	0.79 (0.34-1.82)	0.58	0.88 (0.43-1.82)	0.74
Site				
Manaus	**Ref.**	—	**Ref.**	—
Rio de Janeiro	1.54 (0.74-3.20)	0.25	0.66 (0.30-1.44)	0.30
Salvador	7.68 (0.96-61.50)	0.05	2.45 (0.22-27.51)	0.47
Baseline CD4 count (per 100 cells/μL)	**1.28 (1.12-1.47)**	**<0.001**	1.03 (0.88-1.20)	0.71
Baseline HIV-1 viral load (log_{10})	**0.57 (0.47-0.71)**	**<0.001**	**0.72 (0.59-0.86)**	**<0.001**
CYP2B6 genotypes				
Normal	**Ref.**	—	**Ref.**	—
Intermediate	2.25 (0.85-5.98)	0.10	1.21 (0.59-2.48)	0.61
Slow	1.46 (0.44-4.89)	0.54	0.54 (0.20-1.45)	0.22

ART: antiretroviral therapy; HR: hazard ratio; aHR: adjusted hazard ratio; 95% CI: 95% confidence interval; Significance level .05. Note: among participants on INSTI-based regimens (N = 100), *UGT1A1* genotyping was performed for 90% (N = 90). Similarly, *CYP2B6* genotyping was performed for 84% (N = 64) of participants on EFZ-based regimens (N = 76).
