# Supplementary material for: Determinants of HIV-1 virologic suppression in HIV-associated tuberculosis in Brazil
Source: Mem Inst Oswaldo Cruz. 2026 Jul 10;121:e250090. doi: 10.1590/0074-02760250090 (PMC13336628; doi:10.1590/0074-02760250090)
Supplement: Peer review history

# Reviewer report: FIRST REVIEW ROUND - REVIEWERS COMMENTS

## REVIEWER #1

Major comment

The paper evaluates the relationship between metabolizing genotypes and HIV virological suppression among TB/HIV patients from a Brazilian cohort. The theme is relevant to the field and contributes to identifying factors that could influence the HIV virological suppression.

## Title:

1. The Title suggests the evidence of associations between pharmacogenomics and HIV-1 virological suppression. Since the results do not confirm that, the title should be revised to prevent misinterpretation of the findings (see comment #12).

## Introduction:

2. I suggest including some detailed information about the influence of TB drugs on HIV treatments and their outcomes, like the influence on virological suppression, in the Introduction and Discussion sections.

## Methods and Results:

3. The socio-economic data gathered during the study should be detailed in the Methods and shown in the Results section (Line 106).

4. The clinical data (CD4 cell count and HIV-1 RNA viral load) were collected at baseline, month 2, and at the end of TB treatment visits, as outlined in the Materials and Methods section (Lines 106-107). However, the results of these measurements are not presented in the manuscript. Additionally, it is unclear during which study visit the viral load (presented as a categorical variable) was evaluated.

5. According to the text, the influence of the baseline HIV viral load and the CD4 cell count on the HIV viral load was not considered in the data analysis. Discussing their impact and explaining why they were not evaluated in the study would enhance the manuscript. If this information is unavailable, it should be clearly stated in the Methods and Results section and addressed in the limitations.

6. The authors should clarify how the limits for viral load suppression categories were defined (Lines 124-127).

7. The unit of measurement for viral load needs to be standardized.

8. If the data were available, the authors could assess changes in the viral load during the follow-up period, as an alternative to categorizing the viral load data. If this approach is not feasible, I recommend a brief explanation in the text to clarify the rationale for choosing the categorization approach.

9. The percentage of participants with the UGT1A1 normal metabolizer genotype differs from that in Table 1 (Line 172).

10. The follow statement must be clarified: "Among them, the ones that received efavirenz-based ART were more likely to be CYP2B6 normal metabolizers (n=8, 44%);" as among the participants that do not achieve viral suppression, the ones on efavirenz-based ART were more likely to be CYP2B6 normal or intermediate metabolizers (Lines 179-180).

11. In Figure 3A and B, the median time-to-suppression for the different CYP2B6 and UGT1A1 genotypes did not correspond with the text (Lines 185-190).

## Discussion:

1. In the discussion section, the authors should include additional references related to HIV treatment outcomes, specifically considering data from the Brazilian population (i.e., https://doi.org/10.1371/journal.pone.0305311; https://doi.org/10.31744/einstein_journal/2023AO0156). This approach would provide more context to the Discussion (Lines 203–204).

2. The second paragraph of the Discussion could be summarized and connected more clearly to the results (Lines 211–226).

3. The authors should avoid using expressions like "higher proportion" when there is no statistically significant difference (Line 227).

4. The authors should consider adding comments regarding the lack of difference in time-to-suppression of viral load between efavirenz and INSTI-based regimens, as this finding was anticipated. Additionally, discussing other factors that can influence HIV viral suppression – such as baseline viral load, clinical stage, socio-economic factors, and treatment adherence – would enhance the Discussion. Since some of this data was available, the authors could clarify why these factors were not included in the statistical analysis and acknowledge this as a limitation.

5. Since the authors evaluated the time to viral load suppression for different CYP2B6 and UGT1A1 genotypes, considering two distinct viral load cut points, they could explore these findings in the discussion section.

6. The authors should acknowledge that factors beyond metabolizing genotypes may influence viral suppression. The study did not assess plasma levels of the antiretrovirals or track the viral load throughout the research period. Additionally, no statistically significant differences were found between metabolizing genotypes and achieving viral load suppression. Therefore, the authors should be more conservative in assuming their findings suggest an association between normal metabolizer genotypes and the likelihood of achieving virological suppression.

7. The text could be improved by being more specific about the statement that "virological suppression was low" (Lines 240-241).

8. The text and references need to be reviewed.

## REVIEWER #2

The study titled "Pharmacogenomic associations with HIV-1 virologic suppression in patients with tuberculosis and HIV" by Ridolfi et al. presents results from a two-year follow-up of 194 people living with HIV and coinfected with tuberculosis. The authors evaluated the impact of pharmacogenetic markers in the CYP2B6 and UGT1A1 genes on virological suppression. I have listed three major comments that should be addressed before the manuscript is considered for publication.

1) The primary outcome investigated was virological suppression, defined as viral loads < 1000 copies/mL. However, the standard definition of viral suppression (undetectable viral load) was used only in a secondary analysis and presented in the supplementary material. This choice should be justified.

2) Multivariate analyses should be conducted to adjust for potential confounders such as sex, age, duration of ART, and genetic ancestry. The effect of pharmacogenetic markers is highly influenced by genetic ancestry, as largely described for CYP2B6 genotypes, including studies of people living with HIV in Brazil. Given that participants were recruited from different Brazilian regions with varying degrees of African and Native American ancestries, the study presents an important opportunity to explore ancestry-specific effects. If genetic ancestry estimates are not available, self-reported ethnicity should be included as a covariate, and the limitations of this approach should be discussed. Additionally, adjustment for the duration of ART before enrollment is essential.

3) The proportion of participants who achieved virological suppression, defined as VL < 1000 copies/mL, was unexpectedly low, even for those on dolutegravir-based regimens. The authors should discuss these findings, as they may reflect suboptimal adherence to ART.

## Minor comments

4) In the abstract, please revise the sentence describing the follow-up period. It currently suggests that data analysis was conducted two weeks after ART initiation, whereas the results cover the full two-year follow-up period.

5) Figure 1 indicates that individuals who never initiated ART were excluded from the study. Please clarify whether only those who remained ART-naïve throughout the entire follow-up were removed, or if individuals who were ART-naïve at baseline were also excluded. If the latter, the statistical analysis section should be revised to reflect this. Additionally, the flow diagram does not indicate the period considered for analysis. The abstract mentions a two-week interval, while the methods describe a two-year follow-up. The results support the two-year period.

6) Figures: please revise the legend of the y-axis, which is not in %.

## AUTHORS' RESPONSE TO THE REVIEWERS

Response to Reviewers RE: Manuscript ID MIOC-2025-0090 – "Pharmacogenomic associations with HIV-1 virologic suppression in patients with tuberculosis and HIV" Memórias do Instituto Oswaldo Cruz.

We sincerely thank the reviewers for their thorough evaluation and constructive feedback regarding our manuscript. Based on the reviewers' insightful comments, we have revisited our dataset, included additional variables, and made substantial revisions to the analysis. These changes resulted in new and significant findings, which required substantial rewriting of the Results and Discussion sections. We have provided the tables and figures in a separate file for reviewer's convenience. We believe these updates have greatly enhanced the rigor and scientific contribution of our manuscript. Below, we provide detailed responses to each of the reviewers' comments. Please do not hesitate to contact us if we can provide any additional information.

Sincerely, Felipe Ridolfi, M.D., PhD.

## 1 Reviewer Comments

Reviewer: 1

**Title: 1.** The Title suggests the evidence of associations between pharmacogenomics and HIV-1 virological suppression. Since the results do not confirm that, the title should be revised to prevent misinterpretation of the findings (see comment #12).

• We have revised and changed the title to "Determinants of HIV-1 virologic suppression in HIV-associated tuberculosis in Brazil"

**Introduction: 2.** I suggest including some detailed information about the influence of TB drugs on HIV treatments and their outcomes, like the influence on virological suppression, in the Introduction and Discussion sections.

• A paragraph on this topic has been added to the Introduction, paragraph 2, and in the Discussion section, paragraph 2.

**Methods and Results: 3.** The socio-economic data gathered during the study should be detailed in the Methods and shown in the Results section (Line 106).

• We have updated the relevant sections; Methods, paragraph 3-4, and Results, paragraph 1 and Table 1.

**4.** The clinical data (CD4 cell count and HIV-1 RNA viral load) were collected at baseline, month 2, and at the end of TB treatment visits, as outlined in the Materials and Methods section (Lines 106-107). However, the results of these measurements are not presented in the manuscript. Additionally, it is unclear during which study visit the viral load (presented as a categorical variable) was evaluated.

• We have included additional clinical data for CD4 cell count and HIV-1 RNA viral load. Additionally, we have clarified that the CD4 cell count and viral load included in the Cox regression analysis were those from baseline. Method, paragraphs 4 and 7. Results, all paragraphs.

**5.** According to the text, the influence of the baseline HIV viral load and the CD4 cell count on the HIV viral load was not considered in the data analysis. Discussing their impact and explaining why they were not evaluated in the study would enhance the manuscript. If this information is unavailable, it should be clearly stated in the Methods and Results section and addressed in the limitations.

• We have updated the analysis and included the baseline HIV viral load and CD4 cell count in the uni- and multivariate Cox regression analyses – both were retained as significantly associated with the hazard of virologic suppression. Method, paragraphs 4 and 7. Results, all paragraphs. Discussion, paragraphs 1-6.

**6.** The authors should clarify how the limits for viral load suppression categories were defined (Lines 124-127).

• We chose the definition of virologic suppression as viral load ≤1,000 copies/mL for the primary analysis to align with the WHO guideline, which uses this threshold to define virologic suppression. This approach allowed us to capture more events of virologic suppression, providing sufficient statistical power to identify factors associated with the endpoint of interest. The standard definition of undetectable viral load (≤50 copies/mL) was included in the secondary analysis, and presented in the supplementary material for completeness. We have clarified this rationale in the revised manuscript and included the relevant WHO reference to support this decision. Methods, paragraph 4.

**7.** The unit of measurement for viral load needs to be standardized.

• We have standardized it (copies/mL), throughout the manuscript.

**8.** If the data were available, the authors could assess changes in the viral load during the follow up period, as an alternative to categorizing the viral load data. If this approach is not feasible, I recommend a brief explanation in the text to clarify the rationale for choosing the categorization approach.

• The interpretation of longitudinal viral load patterns in our cohort was limited by the high variability in the timing and number of viral load measurements across participants, a result of routine clinical practice. Because of these factors, the dataset does not meet the assumptions required for reliable modeling of repeated continuous viral load values, and any formal longitudinal analysis would risk producing biased or uninterpretable estimates. For this reason, time-to-virologic suppression was selected as the most robust and clinically meaningful metric, as it accommodates irregular testing schedules and allows for valid comparison across participants with differing follow-up patterns. We have highlighted it in the limitations, Discussion, paragraph 6.

**9.** The percentage of participants with the UGT1A1 normal metabolizer genotype differs from that in Table 1 (Line 172).

• The numbers differ because all participants were genotyped for both UGT1A1 and CYP2B6, but the metabolizer profiles are only relevant for participants who used the corresponding antiretroviral drugs. UGT1A1 metabolizer status applies only to those receiving INSTI-based regimens, while CYP2B6 metabolizer status applies only to efavirenz users. Therefore, although the entire cohort was genotyped, the genotype specific analyses were restricted to the drug-appropriate subgroups. The numbers are different between Table 1, Table 3 (legend), and Figure 3 (legend).

**10.** The follow statement must be clarified: "Among them, the ones that received efavirenz-based ART were more likely to be CYP2B6 normal metabolizers (n=8, 44%);" as among the participants that do not achieve viral suppression, the ones on efavirenz-based ART were more likely to be CYP2B6 normal or intermediate metabolizers (Lines 179-180).

• The text has been revised accordingly.

**11.** In Figure 3A and B, the median time-to-suppression for the different CYP2B6 and UGT1A1 genotypes did not correspond with the text (Lines 185-190).

• We reviewed the data, corrected the information, and updated the figures.

**Discussion: 1.** In the discussion section, the authors should include additional references related to HIV treatment outcomes, specifically considering data from the Brazilian population. This approach would provide more context to the Discussion (Lines 203–204).

• We have included the reference in Discussion Paragraph 2 and 3, respectively.

**2.** The second paragraph of the Discussion could be summarized and connected more clearly to the results (Lines 211–226).

• We have made the requested changes. Discussion, paragraph 1-2.

**3.** The authors should avoid using expressions like "higher proportion" when there is no statistically significant difference (Line 227).

• We have changed the language accordingly.

**4.** The authors should consider adding comments regarding the lack of difference in time-to-suppression of viral load between efavirenz and INSTI-based regimens, as this finding was anticipated. Additionally, discussing other factors that can influence HIV viral suppression – such as baseline viral load, clinical stage, socio-economic factors, and treatment adherence – would enhance the Discussion. Since some of this data was available, the authors could clarify why these factors were not included in the statistical analysis and acknowledge this as a limitation.

• We have made the requested changes. Discussion, paragraph 1-5.

**5.** Since the authors evaluated the time to viral load suppression for different CYP2B6 and UGT1A1 genotypes, considering two distinct viral load cut points, they could explore these findings in the discussion section.

• We have made the requested changes. Discussion, paragraph 4-5.

**6.** The authors should acknowledge that factors beyond metabolizing genotypes may influence viral suppression. The study did not assess plasma levels of the antiretrovirals or track the viral load throughout the research period. Additionally, no statistically significant differences were found between metabolizing genotypes and achieving viral load suppression. Therefore, the authors should be more conservative in assuming their findings suggest an association between normal metabolizer genotypes and the likelihood of achieving virological suppression.

• We have changed the language accordingly. Results, paragraphs 3 and 5. Discussion, paragraphs 5 and 6. As well as conclusions and the Abstract.

**7.** The text could be improved by being more specific about the statement that "virological suppression was low" (Lines 240-241).

• We have addressed this concern. Discussion, paragraphs 1 and 2.

**8.** The text and references need to be reviewed.

• We have thoroughly reviewed the text and updated the references.

## Reviewer: 2

**1.** The primary outcome investigated was virological suppression, defined as viral loads < 1000 copies/mL. However, the standard definition of viral suppression (undetectable viral load) was used only in a secondary analysis and presented in the supplementary material. This choice should be justified.

• We chose the definition of virologic suppression as viral load ≤1,000 copies/mL for the primary analysis to align with the WHO guideline, which uses this threshold to define virologic suppression. This approach allowed us to capture more events of virologic suppression, providing sufficient statistical power to identify factors associated with the endpoint of interest. The standard definition of undetectable viral load (≤50 copies/mL) was included in the secondary analysis, and presented in the supplementary material for completeness. We have clarified this rationale in the revised manuscript and included the relevant WHO reference to support this decision. Methods, paragraph 4.

**2)** Multivariate analyses should be conducted to adjust for potential confounders such as sex, age, duration of ART, and genetic ancestry. The effect of pharmacogenetic markers is highly influenced by genetic ancestry, as largely described for CYP2B6 genotypes, including studies of people living with HIV in Brazil. Given that participants were recruited from different Brazilian regions with varying degrees of African and Native American ancestries, the study presents an important opportunity to explore ancestry-specific effects. If genetic ancestry estimates are not available, self-reported ethnicity should be included as a covariate, and the limitations of this approach should be discussed. Additionally, adjustment for the duration of ART before enrollment is essential.

• We conducted both univariate and multivariate Cox regression analyses to evaluate factors associated with virologic suppression for both the primary endpoint (≤1,000 copies/mL) and the secondary endpoint (≤50 copies/mL). Self-reported race was included in the analyses; however, it was not found to be a significant factor in the univariate or for both endpoints – and because of this finding, it was not included in the multivariate analysis. Additionally, while genetic ancestry was not directly included in this study, our group recently had a related paper accepted for publication in Human Genetics (pre-print available), which evaluated the association between genetic ancestry and treatment outcomes in the RePORT Brazil cohort. That study explored the relationship between genetic ancestry, TB-related adverse drug reactions, and interactions with HIV, providing context for ancestry-specific effects. We have also adjusted for the duration of ART before TB treatment initiation in the multivariate analysis to account for its potential confounding effect. These details have been clarified in the revised manuscript, along with a discussion of the limitations regarding ancestry data. Methods, paragraph 8. Results, paragraph 5. Discussion section thoroughly.

**3)** The proportion of participants who achieved virological suppression, defined as VL < 1000 copies/mL, was unexpectedly low, even for those on dolutegravir-based regimens. The authors should discuss these findings, as they may reflect suboptimal adherence to ART.

• We have addressed this concern in Discussion, paragraph 5. We also added adherence information in Methods, paragraph 3.

**Minor comments 4)** In the abstract, please revise the sentence describing the follow-up period. It currently suggests that data analysis was conducted two weeks after ART initiation, whereas the results cover the full two-year follow-up period.

• We have revised and re-written the whole Abstract.

**5)** Figure 1 indicates that individuals who never initiated ART were excluded from the study. Please clarify whether only those who remained ART-naïve throughout the entire follow-up were removed, or if individuals who were ART-naïve at baseline were also excluded. If the latter, the statistical analysis section should be revised to reflect this. Additionally, the flow diagram does not indicate the period considered for analysis. The abstract mentions a two-week interval, while the methods describe a two-year follow-up. The results support the two-year period.

• Participants who never initiated or used any ART regimen at any point during the study were excluded, as our analysis specifically aimed to evaluate the impact of genetic polymorphisms on ART metabolism and virologic suppression. Individuals who were ART-naïve only at baseline but initiated ART during follow-up were included in the study. This has also been clarified in both the Abstract and Methods, paragraph 4.

**6)** Figures: please revise the legend of the y-axis, which is not in %.

• We have revised and corrected the figures. References 1. 2. World Health Organization. Consolidated guidelines on the use of antiretroviral drugs for treating and preventing HIV infection: recommendations for a public health approach – 2nd ed [Internet]. Geneva; 2016. 480 p. Available from: https://www.who.int/publications/i/item/9789241549684 Piekos JA, Amorim G, Ridolfi F, Cordeiro-Santos M, Kritski AL, Figueiredo MC, et al. Genetic ancestry proportion influences risk of adverse events from tuberculosis treatment in Brazil. medRxiv. 2024 Aug 30;2024.08.29.24312595.

# Reviewer report: HANDLING EDITOR’S COMMENTS

## Dear Dr. Ridolfi and Colleagues,

Thank you for your diligent work in revising the manuscript titled "Determinants of HIV-1 virologic suppression in HIV-associated tuberculosis in Brazil." We appreciate the substantial efforts you have undertaken to address the initial round of reviews. The revisions, including the title change, the addition of clinical data (baseline CD4 and viral load) to the analyses, the standardization of viral load units, and the expansion of the Introduction and Discussion with context regarding the Brazilian population and drug interactions, have significantly improved the clarity and depth of the manuscript.

The revised manuscript has been re-evaluated by our reviewers. While they agree that the previous concerns have largely been met, a few critical issues remain that must be addressed to finalize the manuscript. We ask that you attend to the following points:

## 1. Correction of Supplemental Figure 1 (Reviewer 1)

The reviewers noted a technical error in the supplementary materials. Supplemental Figure 1 currently appears to be identical to Figure 1, including the figure title, despite the legend referring to the secondary analysis. Please correct this file to accurately reflect the data for the secondary endpoint (≤50 copies/mL) and ensure the legend is consistent with the visual content.

## 2. Adjustment for Study Site Heterogeneity (Reviewer 2)

A major methodological and interpretative issue requires correction. As presented in Table 1, there is a stark disparity in virologic suppression rates between the study sites (e.g., approximately 83% suppression in Rio de Janeiro vs. ~39% in Manaus). This substantial site-level variation is a potential confounder that is not accounted for in the main Cox regression models presented in Tables 2 and 3.

**Required Action in Methods:** Please modify the Statistical Analysis section to state that "Study Site" was included as a covariate in the multivariate Cox proportional hazards models. This adjustment is essential to ensure that the reported hazard ratios for other variables (e.g., genotype, baseline CD4) are not confounded by regional differences in healthcare access, implementation of Directly Observed Therapy (DOT), or socio-economic factors.

**Required Action in Results:** Please update the results text and Tables 2 and 3 (and corresponding Supplemental Tables) to present the hazard ratios and confidence intervals associated with "Study Site" after adjustment.

**Required Action in Discussion:** The Discussion section currently overlooks this regional disparity. Please add a paragraph to discuss the observed difference in suppression rates between Rio de Janeiro and Manaus. We encourage you to explore potential reasons for this inequity (e.g., healthcare infrastructure, adherence support, patient demographics) and discuss the implications of these findings for TB/HIV treatment policies in Brazil.

We believe that addressing these specific points will ensure the statistical robustness of your conclusions and maximize the public health impact of your study. We look forward to receiving your revised manuscript.

## AUTHORS' RESPONSE TO THE REVIEWERS

Response to Reviewers RE: Manuscript ID MIOC-2025-0090.R1 – "Determinants of HIV-1 virologic suppression in HIV-associated tuberculosis in Brazil" Memórias do Instituto Oswaldo Cruz. We thank the reviewers for their thorough evaluation and constructive feedback regarding our manuscript.

Based on the reviewers' comments, we have performed additional statistical analysis to account for site heterogeneity (including site in the uni- and multivariate analysis), revised the manuscript thoroughly, and updated the tables and figures. These changes have improved statistical robustness, and we believe have maximized the impact of our study. We have provided tracked-changes and clean versions of the manuscript, and updated tables and figures in a separate file for reviewer's convenience. Below, we provide detailed responses to each of the reviewers' comments.

Please do not hesitate to contact us if we can provide any additional information.

Sincerely, Felipe Ridolfi, M.D., PhD.

## Reviewer Comments & Suggestions

**1. Correction of Supplemental Figure 1.** The reviewers noted a technical error in the supplementary materials. Supplemental Figure 1 currently appears to be identical to Figure 1, including the figure title, despite the legend referring to the secondary analysis. Please correct this file to accurately reflect the data for the secondary endpoint (≤50 copies/mL) and ensure the legend is consistent with the visual content.

Re: We have corrected the figure and its title accordingly.

**2. Adjustment for Study Site Heterogeneity & Adjustment in Methods.** A major methodological and interpretative issue requires correction. As presented in Table 1, there is a stark disparity in virologic suppression rates between the study sites (e.g., approximately 83% suppression in Rio de Janeiro vs. ~39% in Manaus). This substantial site-level variation is a potential confounder that is not accounted for in the main Cox regression models presented in Tables 2 and 3. Please modify the Statistical Analysis section to state that "Study Site" was included as a covariate in the multivariate Cox proportional hazards models. This adjustment is essential to ensure that the reported hazard ratios for other variables (e.g., genotype, baseline CD4) are not confounded by regional differences in healthcare access, implementation of Directly Observed Therapy (DOT), or socio-economic factors.

Re: we have included site in the univariate analysis and as a covariate in the multivariate analysis. For the primary endpoint (viral load ≤1,000 copies/mL) and setting Manaus as the reference category, participants from Rio de Janeiro site had higher hazards of virologic suppression (HR: 1.49, 95% CI: 1.02–2.17). This association, however, was not maintained in the multivariate analysis, suggesting that site did not exert an independent effect on virologic suppression. We have updated the Methods section, lines 185-189; Results section, lines 206-208, lines 242-244; added a paragraph with references in the Discussion section, lines 283-290; and updated the tables accordingly.

**3. Required Action in Results.** Please update the results text and Tables 2 and 3 (and corresponding Supplemental Tables) to present the hazard ratios and confidence intervals associated with "Study Site" after adjustment.

Re: we have updated the Tables and corresponding Supplemental files according to the updated Cox multivariate regression models, which now include site as a covariate.

**4. Required Action in Discussion.** The Discussion section currently overlooks this regional disparity. Please add a paragraph to discuss the observed difference in suppression rates between Rio de Janeiro and Manaus. We encourage you to explore potential reasons for this inequity (e.g., healthcare infrastructure, adherence support, patient demographics) and discuss the implications of these findings for TB/HIV treatment policies in Brazil.

Re: a paragraph exploring the difference in virologic suppression according to sites was added in the Discussion section, lines 283-290, with references.

# Reviewer report: HANDLING EDITOR’S COMMENTS

## Editorial Decision Letter

Manuscript ID: MIOC-2025-0090.R2

Title: Determinants of HIV-1 virologic suppression in HIV-associated tuberculosis in Brazil

Dear Dr. Ridolfi and colleagues,

I have reviewed the revised version of your manuscript (R2) alongside the response to the reviewers.

## Evaluation of Revisions:

The authors have satisfactorily addressed the major concerns raised in the previous round of review. Specifically:

1. **Adjustment for Site Heterogeneity:** The authors have correctly included "study site" as a covariate in the multivariate Cox regression models, as requested by Reviewer 2. The Results section now clearly reports the univariate hazard ratios for the Rio de Janeiro site and correctly notes the lack of independent effect in the multivariate model.

2. **Discussion of Regional Disparity:** The new paragraph in the Discussion section (lines 285-295) effectively addresses the regional differences between Rio de Janeiro and Manaus. The explanation regarding the clinical complexity and referral patterns in Manaus provides necessary context for the observed disparities in suppression rates.

3. **Correction of Supplemental Figure 1:** The authors have confirmed the correction of the figure duplication in the response letter.

The scientific content of the manuscript is now robust and suitable for publication. However, the manuscript text provided appears to contain editing artifacts (likely from a "tracked changes" view) that obscure the numerical data in the Results section. Additionally, the header contains duplicate information regarding word counts and references. These formatting issues must be resolved before final publication.

## Required Minor Corrections (Formatting and Text Cleaning):

Please implement the following corrections to ensure the text is clean and accurate.

**1. Header Section (Lines 26-30)** The header currently contains duplicate lines for word count and references.

**2. Results Section (Line 219)** Substitute the phrase: "median time-to-virologic suppression, irrespective of ART regimen, was 84 (95%CI Interquartile range [IQR] 42-125) days" by: "median time-to-virologic suppression, irrespective of ART regimen, was 84 days (IQR: 42–125)"

**3. Results Section (Line 223)** Substitute the phrase: "median days: 32 6 vs 147 64 days, log-rank χ²=15.1, p<0.001 χ²= 48.5, p<0.001" by: "median days: 6 vs 64 days (log-rank χ²= 48.5, p<0.001)"

**4. Results Section (Lines 226-227)** Substitute the phrase: "log-rank χ²=75.9 12.6 and 29.6 5.02, respectively, with p<0.001 and p-0.02, respectively for both" by: "log-rank χ²=12.6 and 5.02, respectively, with p<0.001 and p=0.02, respectively"

**5. Results Section (Line 230)** Substitute the phrase: "65 57 days (IQR 95% CI: 13–117 54-93)" by: "57 days (IQR: 54–93)"

**6. Results Section (Lines 231-232)** Substitute the phrase: "67 62 days (IQR 95% CI: 19–115 38-153)" by: "62 days (IQR: 38–153)"

**7. Results Section (Lines 232-233)** Substitute the phrase: "147 75 days (IQR 95% CI: 29–265 53-196) (log-rank χ²==2.53, p=0.283 0.57, p=0.753)" by: "75 days (IQR: 53–196) (log-rank χ²=0.57, p=0.753)"

**8. Results Section (Lines 235-238)** Substitute the phrase: "118 60 days (IQR 95% CI: 17–219 27-177)... 58 57 days (IQR 95% CI: 48–68 14-160)... 4 30 days (IQR 95% CI: 0–89) for those with slow genotypes (log-rank χ²=0.07, p=0.967 χ²=1.5, p=0.48)" by: "60 days (IQR: 27–177)... 57 days (IQR: 14–160)... 30 days (IQR: 0–89) for those with slow genotypes (log-rank χ²=1.5, p=0.48)"

**9. Results Section (Line 242)** Substitute the phrase: "HR: 1.12 95% CI 1.11-1.30" by: "HR: 1.12, 95% CI: 1.01–1.30"

Once these text-cleaning corrections are made, the manuscript will be ready for publication.

Sincerely,

Marcelo Ribeiro Alves

Head Editor

*Memórias do Instituto Oswaldo Cruz*
